# Activating transcription factor 3 represses inflammatory responses by binding to the p65 subunit of NF-κB

**DOI:** 10.1038/srep14470

**Published:** 2015-09-28

**Authors:** Ji-Woong Kwon, Hyuk-Kwon Kwon, Hyeon-Jun Shin, Yong-Min Choi, Muhammad Ayaz Anwar, Sangdun Choi

**Affiliations:** 1 Department of Molecular Science and Technology, Ajou University, Suwon, 443-749, Korea

## Abstract

Activating transcription factor 3 (ATF3) is induced by inflammatory responses, cell death, cytokines, and oxidative stress conditions. ATF3 is a negative regulator in the Toll-like receptor 4 signalling pathway. The principal molecule in this pathway is nuclear factor κB (NF-κB) that translocates into the nucleus to initiate the transcription of inflammatory mediators. However, scarce data are available regarding the interaction of ATF3 and p65, a part of the NF-κB dimer. Therefore, we studied the mechanism of regulation of p65 by ATF3 in RAW 264.7 cells. First, LPS-mediated NF-κB activation was confirmed, and then the direct interaction of ATF3 and p65 was observed through immunoprecipitation (IP). The presence of histone deacetylase 1 (HDAC1) was also detected in the complex. In ATF3 deficient cells, NF-κB activity was up-regulated and HDAC1 was not detected by IP. These observations suggest that p65 is attenuated by ATF3 such that ATF3 recruits HDAC1 to the ATF3/p65 complex and facilitates the deacetylation of p65. Likewise, inflammatory response genes were induced by translocated NF-κB in ATF3-deficient cells. Cumulatively, we uncovered a novel mechanism for the negative regulation of NF-κB by ATF3 via direct interaction with p65.

Toll-like receptors (TLRs) are categorized as pattern recognition receptors usually expressed in sentinel cells including antigen-presenting cells. Activation of TLR signalling pathways that converge on nuclear factor κB (NF-κB) trigger the production of pro-inflammatory cytokines and initiate antigen presentation functions^1^,^2^,^3^. Because, TLRs can stimulate the immune system, their ligands are useful in treating a variety of diseases, including cancers^4^,^5^,^6^.

NF-κB plays significant roles in biological responses such as immunity, cell proliferation, inflammation, differentiation, and apoptosis^7^. The mammalian NF-κB family is composed of RelA (p65), c-Rel, RelB, p50, and p52, which form homo- or heterodimers at the κB sites of the promoters of target genes. Whereas RelA/c-Rel, RelA/p50, RelB/p50, and RelB/p52 heterodimers are transcriptional activators, homodimers such as p50/p50 and p52/p52 generally behave as transcriptional repressors^8^,^9^,^10^. For normal physiology, regulated NF-κB-induced inflammation is desirable and is achieved through diverse mechanisms of post-translational modification, ubiquitination of various components, deacetylation of promoter sites, and the translocation of NF-κB into cytosol. The dysregulation of NF-κB-induced inflammation induces human disorders including inflammatory and neurodegenerative diseases^11^ and various cancers^12^,^13^,^14^.

NF-κB is normally held in an inactive form in the cytosol by inhibitor of κB (IκB) proteins^15^,^16^. Various members of the IκB family target different NF-κB dimers, e.g., IκB-α and IκB-β preferentially interact with RelA (p65)/p50 and c-Rel/p50 heterodimers, respectively, IκB-∊ binds only with p65 and c-Rel hetero- and homodimers^17^,^18^,^19^, and Bcl-3 associates only with p50/p50 or p52/p52 homodimers^20^. Although various IκB proteins interact with the same NF-κB homo- or heterodimers, the transcription factors are regulated differentially owing to functional differences among the proteins. For example, IκB-α is rapidly degraded in stimulated cells, resulting in immediate release of NF-κB, but IκB-β degrades over a longer time, causing sustained activation of transcription factors^21^,^22^.

Activating transcription factor 3 (ATF3) is a member of the ATF/cyclic adenosine monophosphate response element–binding (CREB) protein family of bZip transcription factors that binds to the consensus cyclic adenosine monophosphate response element site^23^,^24^. ATF3 functions as a transcriptional repressor by forming a homodimer^25^. In macrophages, ATF3 is increased by lipopolysaccharide (LPS; a TLR4 ligand), bacillus Calmette-Guérin^26^,^27^, and interferon in human peripheral blood mononuclear cells. Moreover, ATF3 is also up-regulated by TLR4 signalling and is part of the negative feedback loop that regulates the LPS-stimulated inflammatory response^22^.

Formerly, ATF3 was thought to deacetylate κB sites in the promoter of target genes by recruiting histone deacetylase 1 (HDAC1) and attenuating cytokine release^28^. However, it was unknown whether ATF3 directly interacts with p65 to regulate its activity. Therefore, this study was designed to explore the underlying mechanism of the negative regulation of p65 in TLR4 signalling. This study revealed the novel perspective that ATF3 not only attenuates the promoter binding of NF-κB but also inhibits NF-κB activity through physical association, which may help modulate the TLR4 signalling pathway in some diseases.

## Results

### LPS-induced inflammatory responses in RAW 264.7 cells

**Figure 1:**
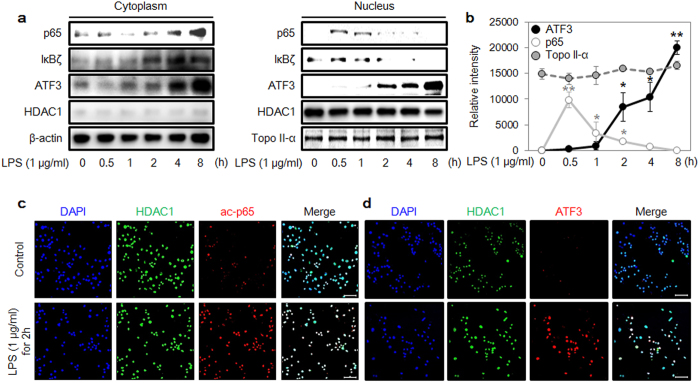
Sensitization of cells to lipopolysaccharide (LPS)-induced inflammatory responses. (**a**) LPS (1 μg/mL)-mediated induction of p65, IκBζ, ATF3, β-actin, HDAC1 and topoisomerase II-α protein expression in cytoplasm and nucleus. RAW 264.7 cells were treated with 1 μg/mL LPS for the indicated times. Cell lysates were processed with NE-PER lysate, and the protein expression levels were measured with western blot. β-actin was used as a loading control in cytoplasm. HDAC1 and topoisomerase II-α were used as loading controls in nucleus. (**b**) Intensities of ATF3, p65, and topoisomerase II-α for the indicated times, calculated using ImageJ software. Data represent three independent experiments (*n* = 3; *P < 0.05, **P < 0.01). (**c**,**d**) Levels of acetylated p65 (ac-p65), ATF3, and HDAC1 proteins in nucleus were measured with confocal microscopy. Nuclei were stained with Hoechst stain (blue color). Scale bar: 20 μm.

First, we treated RAW 264.7 cells with LPS and observed the translocation of various proteins into the nucleus using Western blot (Fig. 1a). ATF3 expression was rapidly up-regulated after 2 h, whereas the amount of HDAC1 in the nucleus was unaffected by LPS treatment. Previous reports have shown that ATF3 is a negative regulator that binds to the promoter region of target genes and recruits HDAC1 to form a complex to modulate inflammation^28^. The p65 protein rapidly accumulated in the nucleus within 30 min, induced the expression of target genes for 2 h, and then vanished. The level of ATF3 corresponded inversely with the level of p65 protein in the nucleus, likely because the accumulating ATF3 decreased the nuclear concentration of p65 (Fig. 1b). Moreover, the results from confocal microscopy showed that LPS treatment for 2 h increased acetylated p65 (ac-p65) and induced the translocation of ATF3 into the nucleus (Fig. 1c,d).

### Direct binding and regulation of p65 by ATF3

**Figure 2:**
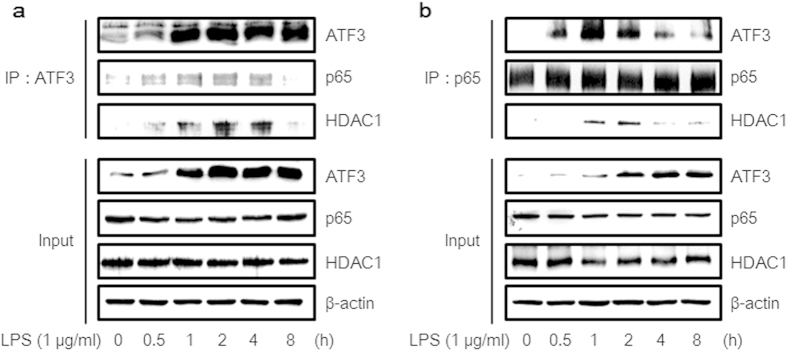
ATF3 interacts with the p65 subunit of nuclear factor κB (NF-κB). (**a**) Cells were treated with 1 μg/mL LPS for the indicated times. Beads were labelled with ATF3 antibody, and the interacting proteins, which were bound to ATF3, were precipitated. A total of 20 μg protein was separated with electrophoresis. The levels of these proteins were determined with Western blot using whole-cell lysates. (**b**) Cells were treated with 1 μg/mL LPS for the indicated times. Beads were labelled with p65 antibody, and the proteins bound to p65 were precipitated. A total of 20 μg protein was separated with electrophoresis. Protein levels were determined with Western blot using whole-cell lysates. Data show a representative set among the results of three independent experiments (*n* = 3).

Using previous observations, we hypothesized that ATF3 is associated with p65 activation. Therefore, we treated cells with LPS in a time-dependent manner and performed immunoprecipitation (IP) to decipher the interaction of ATF3 with p65. In several independent experiments, antibodies specific to either ATF3 or p65 were agarose bead-labelled and used to extract the cognate protein bound to the labelled protein. In anti-ATF3 antibody beads, we observed p65 bound to ATF3 in as quickly as 30 min (Fig. 2a). HDAC1 associated with ATF3 could bind at the target promoter region and inhibit target gene expression by deacetylating the promoter and was bound with ATF3 at 30 min. Moreover, when anti-p65 antibody was attached to the agarose beads and the complex was extracted, we observed that the ATF3/p65 complex formed with the same binding kinetics (Fig. 2b). These results supported our hypothesis that ATF3 physically interacts with p65. Furthermore, these results could be corroborated the hypothesis that the reduction of p65 protein in the nucleus after 30 min was correlated with ATF3.

### Inhibition of LPS-induced nuclear localization of p65 by ATF3

**Figure 3:**
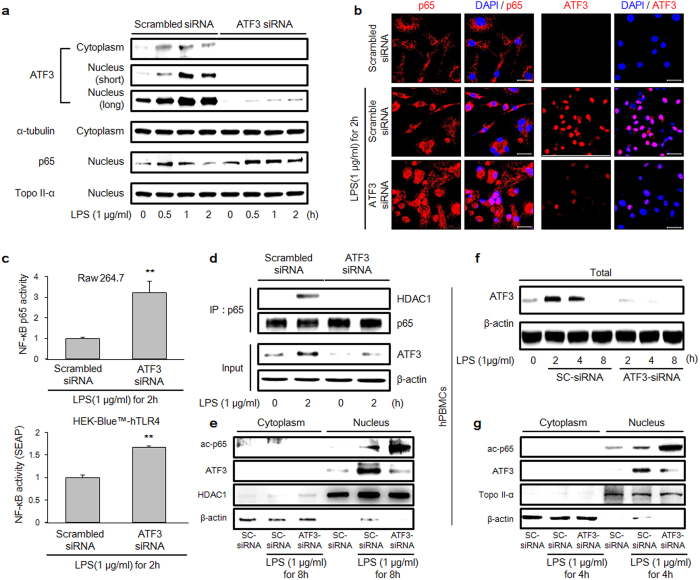
Activation of NF-κB via ATF3 down-regulation. (**a**) Cells were transfected with 100 nM ATF3 small interfering RNA (siRNA) and reagent alone for 48 h and then treated with 1 μg/mL LPS for the indicated times. The protein levels of ATF3, p65, α-tubulin, and topoisomerase II-α were detected with Western blot using the respective antibodies. α-tubulin and topoisomerase II-α were used as a loading control for cytoplasm and nucleus, respectively. (**b**) p65 and ATF3 were assessed with confocal microscopy. Cells were transfected with 100 nM ATF3 siRNA and reagent for 48 h and then treated with 1 μg/mL LPS for 2 h. (**c**) RAW 264.7 cells (left panel) were transfected with ATF3 siRNA and reseeded from a 10-cm dish to a 96-well plate to quantify NF-κB activity using enzyme-linked immunosorbent assay (ELISA). Human embryonic kidney 293 (HEK293) cells (right panel) were also transfected with ATF3 siRNA, and NF-κB activity was detected with SEAP assay using ELISA. Data represent three independent experiments (*n* = 3; *P < 0.05, **P < 0.01). (**d**) Cells were treated with 1 μg/mL LPS for the indicated times. Beads were bound with p65 antibody, and p65 and HDAC1 were precipitated. A total of 20 μg protein was separated with electrophoresis. Proteins levels were determined with Western blot using whole-cell lysates. (**e**) Cell lysates were processed with NE-PER lysate, and the protein expression levels of ATF3, ac-p65, HDAC1 and β-actin were measured by Western blot. β-actin and HDAC1 were used as a loading control for cytoplasm and nucleus, respectively. (**f**,**g**) Human PBMCs were transfected with 100 nM ATF3 siRNA and reagent alone for 48 h and then treated with 1 μg/mL LPS for the indicated times. (**f**) The protein levels of ATF3 and β-actin were detected by Western blot; β-actin was used as a loading control. (**g**) The protein expression levels of ATF3, ac-p65, topoisomerase II-α, and β-actin were measured with Western blot. β-actin and topoisomerase II-α were used as a loading control for cytoplasm and nucleus, respectively. Western data show a representative set among the results of three independent experiments (*n* = 3).

From analyses of previous data, we hypothesized that the translocation of p65 to the nucleus is closely related to ATF3 expression. Therefore, we monitored the activation of p65 in the absence of ATF3 using ATF3 small interfering RNA (siRNA) in TLR4 signalling. In ATF3-deficient RAW 264.7 cells, the up-regulated expression of p65 was maintained from 30 min to 2 h compared with p65 expression in cells treated with scrambled siRNA (Fig. 3a). In addition, we observed ATF3 and p65 in the nucleus after 2 h of LPS treatment using confocal microscopy (Fig. 3b). After 2 h of LPS treatment, ATF3 was detected in the nucleus in scrambled siRNA-treated cells, but p65 was not. However, in ATF3-deficient RAW 264.7 cells, p65 protein was significantly detected in the nucleus with confocal microscopy even after 2 h of LPS treatment, as has been seen in the Western blot results (Fig. 3a). These observations confirmed that the translocation of p65 is associated with ATF3 protein expression.

### Recruitment of HDAC1 by ATF3 to regulate NF-κB activation

We confirmed that the expression of ATF3 protein is associated with the translocation of p65. We also determined whether ATF3 affects the activation of p65. To confirm the down-regulation of NF-κB activity via binding with ATF3, we performed NF-κB activity assays using enzyme-linked immunosorbent assay (ELISA) and secreted embryonic alkaline phosphatase (SEAP). ELISA is a powerful detection method that measures the activity of NF-κB in nuclear extracts. Activated p65 binds to the κB site in DNA, and additional horseradish peroxidase-conjugated secondary antibody reacts with the substrate. When the expression of ATF3 was inhibited using siRNA in RAW 264.7 cells, NF-κB activity was up-regulated threefold to that of the control in response to LPS (Fig. 3c, top). SEAP assay detects NF-κB activity using the κB site of immune response target genes^29^. The deacetylated-p65, which was ATF3-assisted deacetylated, was eliminated in the nucleus and could not bind with the κB site of the target genes, thereby resulting in the down-regulation of NF-κB target genes. Using this assay, we found that NF-κB activity was up-regulated 1.5-fold by LPS treatment, whereas ATF3 expression was inhibited by siRNA in human embryonic kidney 293 (HEK293)-Blue TLR4 reporting cells (Fig. 3c, bottom).

In addition, we performed IP under ATF3-deficient conditions (Fig. 3d). After 2 h of LPS treatment, p65 bound with HDAC1 in scrambled siRNA but failed to bind under ATF3-deficient conditions. Moreover, treatment of ATF3-deficient cells with LPS resulted in the increased expression of ac-p65 in the nucleus compared with scrambled siRNA cells at 8 h (Fig. 3e). In human peripheral blood mononuclear cells (hPBMCs), LPS induced the expression of ATF3 at 2 and 4 h that was inhibited by ATF3 siRNA (Fig. 3f). Furthermore, ATF3-deficient cells also showed an increase in the expression of ac-p65 in the nucleus following its treatment with LPS compared to scrambled siRNA cells at 4 h (Fig. 3g). These results showed that ATF3 has critical importance in mediating interaction between p65 and HDAC1, as well as in the acetylation of p65. The reduced acetylation of p65 in ATF3 presence implied an indirect role of ATF3 in governing the acetylation status of p65 that in turn mediates the transcription of inflammatory genes (Fig. 3g).

### ATF3 regulates NF-κB-dependent gene expression

**Figure 4:**
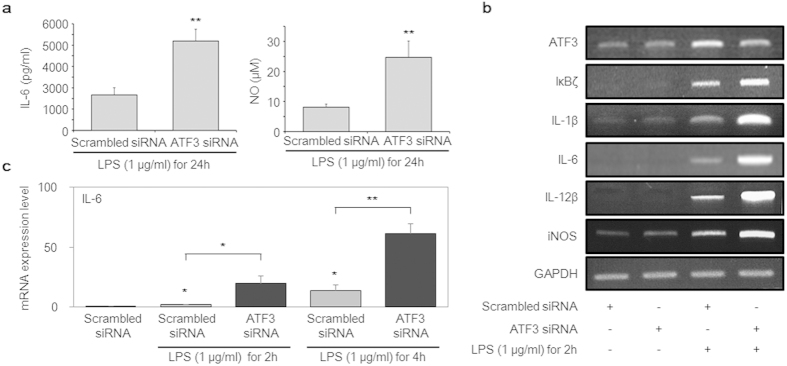
Inflammatory response genes in NF-κB signalling were up-regulated under ATF3-deficient conditions. (**a**) Cells were transfected with 100 nM ATF3 siRNA for 48 h and then treated with 1 μg/mL LPS for 24 h. The secretion of interleukin (IL)-6 and nitric oxide (NO) were detected with ELISA. (**b**) RAW 264.7 cells were transfected with scrambled siRNA or ATF3 siRNA for 48 h and then treated with 1 μg/mL LPS for 2 h. Messenger RNA expression was measured using conventional reverse transcription-polymerase chain reaction. (**c**) RAW 264.7 cells were transfected with scrambled siRNA or ATF3 siRNA for 48 h and then treated with 1 μg/mL LPS for 2 and 4 h. Messenger RNA expression of *IL-6* was measured using real-time polymerase chain reaction. Expression of *IL-6* was normalized to *β-actin* and compared to control cells. Data represent three independent experiments (*n* = 3; *P < 0.05, **P < 0.01).

Interleukin (IL)-6 and nitric oxide (NO) are representative inflammatory response molecules^30^,^31^. NO is produced by inducible NO synthase induced by NF-κB signalling. IL-6 is also induced by NF-κB signalling via binding to the κB site in the IL-6 promoter^30^. Therefore, we confirmed IL-6 and NO expression using ELISA (Fig. 4a) and observed that both were up-regulated under ATF3-deficient conditions. Moreover, a plethora of inflammatory mediators were expressed, including IκBζ, IL-1β, IL-6, IL-12β, and inducible NO synthase when NF-κB was activated. It has been reported that ATF3 can bind to the IL-6 and IL-12β promoter regions and inhibit transcription via deacetylation of the κB site^28^.

Furthermore, we evaluated messenger RNA expression of the target genes of well-known primary inflammatory mediators using reverse transcription-polymerase chain reaction (RT-PCR) after treating cells with LPS for 2 h (Fig. 4b). As the results indicated, these genes were up-regulated compared with those in the control. Of particular interest, the expression level of *IL-6* was significantly altered in ATF3-deficient cells compared with scrambled siRNA cells in a time-dependent manner (Fig. 4c). Cumulatively, the results of this study showed that ATF3 binds to NF-κB (p65) directly and creates a docking site for HDAC1 to deacetylate p65 to inhibit inflammatory response cytokines induced by NF-κB signalling. Our findings present a mechanism other than the canonical one stating that ATF3 binds directly with the cytokine promoter region, not the NF-κB subunit.

### Discussion

The stress-induced transcription factor ATF3 is a negative regulator of cytokines such as IL-6, IL-12β, and tumour necrosis factor-α in LPS-induced TLR4 signalling^22^,^28^,^32^. NF-κB is the principal mediator of TLR4 signalling that, upon activation, translocates into the nucleus and induces inflammation. *ATF3* is also a target gene of NF-κB and is induced when NF-κB is activated^33^. The canonical mechanism of ATF3-negative regulation proposes that ATF3 recruits HDAC1 to the transcriptionally active complex and assists in deacetylating the promoter region of inflammatory cytokines such as IL-6, thereby impeding p65 binding to the target promoters and in turn attenuating their transcription^22^,^28^. In this study, we demonstrated that NF-κB activity is attenuated via direct interaction of ATF3 and the p65 subunit of NF-κB rather than through an indirect mechanism.

The detection of an immune-activating ligand such as LPS culminated in the activation of the immune system; as a result, inflammatory mediators came into play to combat the invading pathogen. This host–pathogen interaction is tightly regulated so that neither the pathogen harms the organism nor an exuberant immune response yields unwanted consequences^34^. This tight regulation is orchestrated by multiple proteins that execute the response in a coordinated fashion. Similarly, when TLR4 senses LPS, it generates many cytokines (Fig. 1a). This activation also triggers the mechanisms of negative regulation, such as ATF3 translocation into the nucleus. The time frames of p65 degradation and ATF3 accumulation coincide, pointing to the negative influence of ATF3 on p65 (Fig. 1b).

The results of recent studies have suggested that ATF3 recruits HDAC1 to the p65-harbouring transcriptional complex and deacetylates the promoters, resulting in p65 dissociation^28^,^35^,^36^,^37^. Furthermore, these studies have implied that an alternative spliced form of ATF3, ATF3Δzip2, but not ATF3, binds to p65 and modulates the response^35^. In this study, we demonstrated through IP analysis that not only ATF3Δzip2 but also ATF3 can bind to mp65 (Fig. 2a,b). Moreover, p65 binds to HDAC1 (Fig. 2b), but under ATF3-deficient conditions (Fig. 3d), p65 is unable to bind to HDAC1. This intriguing behaviour could be attributed to ATF3 interaction with p65 that creates a docking site for HDAC1. The docked HDAC1 then plausibly deacetylates p65, facilitating its export from the nucleus. Therefore, we now believe that ATF3 is a key molecule in mediating the interaction between p65 and HDAC1 and that it attenuates p65 activity.

To unravel the requirement of ATF3 for the binding of HDAC1 in these interactions, we conducted experiments under ATF3-deficient conditions. We observed that under these conditions, p65 was maintained in the nucleus for a longer time in response to LPS treatment compared to that in the control (Fig. 3a). Additional data showed that NF-κB was significantly up-regulated in cells lacking ATF3 (Fig. 3b,c). The formation of ternary complex can be deduced by a partial direct contribution from each molecule. In the presence of ATF3, HDAC1 bound to p65 was detected (Fig. 2b), whereas in the absence of ATF3, this association was impaired (Fig. 3d), clearly implying the necessary role of ATF3 in mediating this ternary complex. In contrast to those of previous studies, our results revealed a lack of interaction between p65 and HDAC1 in the absence of ATF3, which precluded the deacetylation of p65 and increased p65 activity compared with that in the control (Fig. 3e). The presence of ATF3 suppressed the level of ac-p65, whereas the ATF3-siRNA enhanced the ac-p65 level, implying the ATF3 role in acetylation of p65, and the plausible mechanism is the recruitment of HDAC1 on various complexes that carried out deacetylation of p65. We observed the similar results when the experiments were repeated in hPBMCs, indicating that this might be a canonical mechanism that is followed in various cells (Fig. 3f,g). Therefore, we concluded that ATF3 facilitates the interaction of HDAC1 and p65, leading to reduced p65 activity. When cells are lacking ATF3, p65 has ample time to induce its target genes^11^, in particular, those of inflammation-related cytokines that were also observed (Fig. 4).

ATF3 is a key molecule in the regulation of innate immune response, and it is associated with a variety of immune and inflammatory responses. Most of these responses are triggered by NF-κB activation. Further experiments are needed to unravel the details of this regulatory checkpoint, and this study provides a basis for those studies. Moreover, the results of this study furnish a novel mechanism of innate immune regulation by ATF3 that will be helpful in designing therapeutic interventions for inflammation-related abnormalities.

## Materials and Methods

### Cell cultures

RAW 264.7 (Korean Cell Line Bank, Seoul, Korea) and HEK-Blue™-hTLR4 (Invivogen, San Diego, CA, USA) cell lines were cultured in Dulbecco’s modified Eagle medium (DMEM) with low glucose and DMEM growth medium containing 10% FBS and 1% penicillin/streptomycin (Thermo Scientific, Waltham, MA, USA), respectively. Human PBMCs were purchased from PromoCell (Heidelberg, Germany) and cultured in RPMI1640 medium containing 10% FBS, 1% penicillin/streptomycin (Thermo Scientific) and 1% L-glutamine (Gibco, Grand Island, NY, USA). These cells were incubated in an atmosphere of 5% CO_2_ at 37 °C (Thermo Scientific).

### Confocal microscopy analysis

Cells were fixed by 3.7% formaldehyde (Sigma-Aldrich, St. Louis, MO, USA) for 15 min and permeabilized by 0.2% Triton X-100 for 15 min. Cells were blocked by 5% FBS (Thermo Scientific) in PBS (AMRESCO, Solon, OH, USA) for 1 h and then incubated with primary antibodies (1:1000, 1 h) targeting p65, ATF3 (Santa Cruz Biotechnology, Dallas, TX, USA), ac-p65 (Abcam, Cambridge, MA, USA) or HDAC1 (Millipore, Billerica, MA, USA). Later, cells were incubated with secondary antibodies (1:1000, 1 h) conjugated with Alexa Fluor 488 or 546 (Invitrogen, Carlsbad, CA, USA). Nuclei were stained with Hoechst33258 (5 μM; Sigma-Aldrich) for 15 min. Stained cells were visualized using confocal microscopy (LSM-700, Carl Zeiss Microimaging, Germany) and analyzed by Zen 2009 software.

### RNA isolation, and RT- and qRT-PCR

Total RNA was isolated using an RNeasy mini kit (QIAGEN, Valencia, CA, USA), and the total RNA concentration was detected using a Micro UV-Vis fluorescence spectrophotometer (Malcom, Tokyo, Japan). Total RNA (500 ng) was reverse-transcribed to complementary DNA with Oligo dT using an Accupower RT PreMix complementary DNA kit (Bioneer, Daejeon, Korea). PCR was performed using GoTaq^®^ Green Master Mix (Promega, Madison, WI, USA). The RT-PCR primers were as follows: *IL-1β* F; 5′-GCA ACT GTT CCT GAA CTC AAC T-3′ and R; 5′-ATC TTT TGG GGT CCG TCA ACT-3′; *IL-6*, F; 5′-TGG GGC TCT TCA AAA GCT CC-3′ and R; 5′-AGG AAC TAT CAC CGG ATC TTC AA-3′; *IL-12β*, F; 5′-TGG TTT GCC ATC GTT TTG CTG-3′ and R; 5′-ACA GGT GAG GTT CAC TGT TTC T-3′; *iNOS*, F; 5′-CAG GGA GAA CAG TAC ATG AAC AC-3′ and R; 5′-TTG GAT ACA CTG CTA CAG GGA-3′ and the primers for *ATF3*, *TNF-α*, *IκBζ* and *β-actin* are as described previously^24^. Thermal cycling was carried out with an initial denaturation phase of 10 min at 95 °C, followed by 30 cycles each of 30 sec of denaturation at 95 °C, annealing for 30 sec at 57 °C, and extension for 1 min at 72 °C. Amplification was carried out in a Veriti™ thermal cycler (Applied Biosystems, Carlsbad, CA, USA), and the amplified product was subjected to electrophoresis on 1.5% agarose gel. Gel images were captured by UV transilluminator system (TFX-20M; Vilber-Lourmat, Marne-la-Vallée, France). In qRT-PCR analysis, the expression level of *IL-6* was measured using a Maxima SYBR Green/ROX qPCR master mix (Thermo Scientific) and examined using a qRT-PCR (Bio-Rad Laboratories, Hercules, CA, USA) with primers including *IL-6*, as described previously^38^. The two-step PCR protocol was as follows: 10 min at 95 °C, followed by 30 cycles of denaturing (5 s at 95 °C) and annealing (10 s at 57 °C). Relative *IL-6* level was calculated using the ΔΔCT method, and *IL-6* level was normalized to *β-actin* level.

### Western blot analysis

After treatment, protein was isolated by resuspending cells in M-PER^®^ Mammalian Protein Extraction Reagent (Thermo Scientific) and NE-PER lysate (Thermo Scientific) for immunoprecipitation with protease & phosphatase inhibitor cocktails (Thermo Scientific), incubated for 10 min at 4 °C, and centrifuged at 16,000 × *g* for 10 min to remove cell debris. Extracts were loaded onto 10% polyacrylamide gels and transferred to Hybond-ECL membranes (Amersham, Pollards Wood, UK). Membranes were blocked with 5% skim milk in phosphate-buffered saline containing 0.05% Tween 20 (PBST). The membranes were then incubated with specific primary antibodies diluted with PBST at 4 °C with gentle shaking overnight: p65, ATF3, topoisomerase II-α, IκBζ, α-tubulin and β-actin (Santa Cruz Biotechnology); ac-p65 (Abcam) and HDAC1 (Millipore). After several washings, the membranes were incubated with anti-mouse or anti-rabbit immunoglobulin G peroxidase-conjugated antibody (Thermo Scientific) diluted in PBST (1:1,000) for 2 h. After the membranes were washed several times with PBST, detection was carried out using Pierce ECL Western blotting substrate (Thermo Scientific) and exposure of the blots to a LAS-1000 system (Fuji, Tokyo, Japan). The intensities of protein bands were measured using the ImageJ software (http://rsb.info.nih.gov/ij/)^39^.

### IP analysis

RAW 264.7 cells were seeded at an approximate density of 1 × 10^6^ cells/10-cm dish. After treatment, IP with antibodies specific to ATF3 and p65 (Santa Cruz Biotechnology) was performed. Protein was isolated following the method used for Western blot analysis.

### Quantification of IL-6 and p65 with ELISA

The concentrations of IL-6 and p65 in the supernatant of the culture media were determined using commercially available ELISA kits (eBioscience, San Diego, CA, USA). First, respective cells (5 × 10^5^/well) were dispensed into a 24-well plate and stimulated with LPS (1 μg/ml) for IL-6 at 24 h or p65 at 2 h. Then supernatant was collected and added to the antibody-coated microplate to capture IL-6 and p65. The plates were then maintained at room temperature for 2 h. Next, the plates were washed five times, biotin-conjugated detecting antibody was added to each well, and the plates were further incubated at room temperature for 1 h. After incubation, the plates were washed five times and incubated with avidin–horseradish peroxidase for 30 min. Finally, detection was carried out with 3,3′,5,5′-tetramethylbenzidine solution by measuring absorbance at 450 nm with an ELISA reader and analysed using SoftMax^®^ Pro 5.3 software (Molecular Devices, Sunnyvale, CA, USA).

### SEAP reporter assay

SEAP was detected using QUANTI-Blue™ (Invivogen) according to the manufacturer’s instructions. Briefly, 10 μL of supernatant was collected by centrifuging 1 mL of culture medium at 11,200 × *g*. This supernatant was then added to a 96-well plate and heated to 65 °C for 10 min to deactivate endogenous phosphatases. To each well, 50 μL of 1 × dilution buffer, 100 μL of 1 × assay buffer, 20 μL of 100 mM l-homoarginine, and 20 μL H_2_O were added and incubated at 37 °C for 10 min. Finally, 20 μL of staining solution was added, and optical density was measured at 405 nm. LPS stimulation induced the HEK-Blue™-hTLR4 cells to secret alkaline phosphatase as an indication of TLR4 activity. SEAP activity was quantified using QUANTI-Blue™ by measuring optical density at 620–655 nm with an ELISA reader (Molecular Devices).

### NO assay

RAW 264.7 cells were plated at a density of 1 × 10^6^ cells/mL into 96-well plates, incubated for 12 h, and treated with the LPS for 24 h. Supernatant (100 μL) from the cultured cells was transferred to new 96-well plates and quantified using an NO detection kit according to the manufacturer’s protocol (iNtRON Biotech, Seongnam, Korea). Values were calculated by measuring the absorbance at 540 nm using an ELISA reader (Molecular Devices).

### siRNA design and transfection

siRNA for ATF3 was designed and purchased from Bioneer (Daejeon, Korea) as follows: 5-CTG TGA GAT AAG CGG GAC TCA G-3^40^,^41^. Lipofectamine 2000 transfection reagent (Life Technologies, Grand Island, NY, USA) was used for siRNA delivery. RAW 264.7 cells were seeded at 5 × 10^5^ cells in a 6-cm dish containing 2 mL DMEM with low glucose for 12 h. hPBMCs were seeded at 2 × 10^6^  cells in a 6-cm dish containing 2 mL of RPMI1640 medium for 12 h. Opti-MEM Reduced serum medium (2 mL; Life Technologies) containing 100 nM of siRNA and 20 μL of Lipofectamine 2000 reagent was incubated at room temperature for 15 min and added to each well. After transfection with ATF3 siRNA for 48 h, the medium was replaced with normal medium for further experiments.

### Statistical analysis

The statistical analysis was done by the one-way ANOVA using the SigmaPlot software version 12.0 (Systat Software Inc., San Jose, CA, USA). The data shown are the results of at least three independent experiments and the statistical significance was defined as a P-value of *P < 0.05 or **P < 0.01.

## Additional Information

**How to cite this article**: Kwon, J.-W. *et al.* Activating transcription factor 3 represses inflammatory responses by binding to the p65 subunit of NF-κB. *Sci. Rep.* **5**, 14470; doi: 10.1038/srep14470 (2015).
